# Alterations of long noncoding RNAs and mRNAs in extracellular vesicles derived from the murine heart post‐ischemia–reperfusion injury

**DOI:** 10.1111/jcmm.17617

**Published:** 2022-11-28

**Authors:** Xinyu Ge, Qingshu Meng, Xuan Liu, Jing Liu, Xiaoxue Ma, Shanshan Shi, Mimi Li, Fang Lin, Xiaoting Liang, Xin Gong, Zhongmin Liu, Wei Han, Xiaohui Zhou

**Affiliations:** ^1^ Research Center for Translational Medicine Shanghai East Hospital, Tongji University School of Medicine Shanghai China; ^2^ Shanghai Heart Failure Research Center Shanghai East Hospital, Tongji University School of Medicine Shanghai China; ^3^ Department of Cardiothoracic Surgery Shanghai East Hospital, Tongji University School of Medicine Shanghai China; ^4^ Institute for Regenerative Medicine, Shanghai East Hospital, School of Life Sciences and Technology, Tongji University Shanghai China; ^5^ Department of Heart Failure Shanghai East Hospital, Tongji University School of Medicine Shanghai China; ^6^ Shanghai Institute of Stem Cell Research and Clinical Translation Shanghai China

**Keywords:** extracellular vesicles, ischemia–reperfusion injury, long non‐coding RNA, mRNA, RNA sequencing

## Abstract

Extracellular vesicles (EVs) play important roles in cardiovascular diseases by delivering their RNA cargos. However, the features and possible role of the lncRNAs and mRNAs in cardiac EVs during ischemia–reperfusion (IR) remain unclear. Therefore, we performed RNA sequencing analysis to profile the features of lncRNAs and mRNAs and predicted their potential functions. Here, we demonstrated that the severity of IR injury was significantly correlated with cardiac EV production. RNA sequencing identified 73 significantly differentially expressed (DE) lncRNAs (39 upregulated and 34 downregulated) and 720 DE‐mRNAs (317 upregulated and 403 downregulated). Gene Ontology (GO) and pathway analysis were performed to predict the potential functions of the DE‐lncRNAs and mRNAs. The lncRNA‐miRNA‐mRNA ceRNA network showed the possible functions of DE‐lncRNAs with DE‐mRNAs which are enriched in the pathways of T cell receptor signalling pathway and cell adhesion molecules. Moreover, the expressions of ENSMUST00000146010 and ENSMUST00000180630 were negatively correlated with the severity of IR injury. A significant positive correlation was revealed between TCONS_00010866 expression and the severity of the cardiac injury. These findings revealed the lncRNA and mRNA profiles in the heart derived EVs and provided potential targets and pathways involved in cardiac IR injury.

## 1 INTRODUCTION

Myocardial ischemia–reperfusion (IR) injury is a common cardiac disorder that exists in various clinical scenarios, including heart transplantation, cardiac arrest, percutaneous coronary artery intervention, and cardiac surgery. The reperfusion of ischemic hearts can lead to cardiomyocyte death and further aggravate the ischemia‐induced reduction of cardiac function.^1^ However, the underlying molecular mechanism of IR injury remains poorly understood.

Extracellular vesicles (EVs) are nanosized cell‐derived membranous vesicles that exist in various biological fluids.^2^ Previous studies have demonstrated the vital roles of EVs in numerous biological functions, including cancer progression and metastasis,^3^ wound healing,^4^ angiogenesis^5^ and immunoregulation.^6^ Investigations of the features and functions of EVs from heart tissues may provide novel clues that help elucidate the pathological process of cardiac IR injury. As cell‐to‐cell communication vehicles,^7^ EVs can transfer multiple substances such as proteins, lipids, and RNA species (including miRNAs, circRNAs, lncRNAs, and mRNAs) to recipient cells and regulate their functions.^8^, ^9^ Recently, we confirmed the increased release of EVs in the heart during IR^10^ and elucidated the miRNA profile as well as the function of the miRNA cargo in cardiac EVs on cardiac injury.^11^ However, the feature and mechanism of EV‐packaged lncRNAs and mRNAs in the pathogenesis of cardiac IR injury remain elusive.

Long noncoding RNAs (lncRNAs) are regarded as non‐protein‐coding transcripts larger than 200 nt.^12^ Increasing evidence indicated the link between lncRNAs and cardiovascular diseases.^13^, ^14^, ^15^ It has been reported that a lncRNA termed cardiac autophagy inhibitory factor (CAIF) can attenuate myocardial infarction by blocking p53‐mediated myocardin transcription.^16^ Inhibition of lncRNA‐X‐inactive specific transcript (XIST) and lncRNA‐taurine upregulated gene 1 (TUG1) could improve myocardial IR Injury.^17^, ^18^, ^19^ Moreover, the potential applications of lncRNAs as biomarkers for cardiovascular diseases have been verified in recent studies.^20^, ^21^ These results suggested the importance of lncRNAs in cardiovascular diseases. In the present study, we performed RNA sequencing analysis in EVs derived from the heart tissues post‐IR to identify the profiles of lncRNAs and mRNAs in these IR‐EVs and evaluated the association between the differentially expressed (DE)‐lncRNAs and the severity of IR injury, which indicated that cardiac EVs enriched lncRNA and mRNA may represent important players which contribute to the cardiac IR injury.

## 2 MATERIALS AND METHODS

### 2.1 Animals

Male C57BL/6J mice (10 weeks old, 26 ± 1 g) were purchased from SLAC Laboratory Animal Co., Ltd. Mice were housed in a specific‐pathogen‐free room with constant temperature (23–24°C), humidity (55 ± 5%), and light (12–12 h light–dark schedule), and kept in the solid‐bottom cages of polycarbonate with high‐pressure sterilized corncob on the bottom of the cage. There were at most five mice in each cage. All mice had free access to food and water. The animal procedures were performed following the recommendations of national and international laboratory animal care and use and were approved by the Institutional Animal Care and Use Committee of Tongji University (Number: TJLAC‐018‐030).

### 2.2 Establishment of the murine myocardial IR model

Mice were randomly divided into the sham and IR group using computer‐generated random numbers. Myocardial IR models were established according to our previous study.^10^ Briefly, 1.5% pentobarbital (50 mg/kg body weight, Sigma) was intraperitoneally injected for mouse anaesthesia. Endotracheal intubation and mechanical ventilation were performed and then the chest cavity was opened at the 4th intercostal space. The left anterior descending (LAD) coronary artery was ligated with an 8–0 silk suture. The suture was then removed to allow reperfusion after 45 min (I45minR24h) or 1 h (I1hR24h). After the operation, the mice were placed on a heated blanket until they recovered from anaesthesia. Blood and heart samples were collected 24 h later.

### 2.3 Echocardiography and myocardial enzyme detection

The mice were examined by echocardiography (VisualSonics Inc) 1 day after IR. After depilation, the mice were anaesthetised with isoflurane (1–2% vol/vol, RWD life science) and fixed in a supine position. The left ventricular end‐diastolic diameter (LVEDD) and left ventricular end‐systolic diameter (LVESD) were measured by placing a small probe in the left anterior chest of the mice. The left ventricular ejection fraction (EF) and fraction shortening (FS) were calculated for cardiac function evaluation.

The mice were anaesthetised with 1.5% pentobarbital before blood collection from the mouse eyes. The sera from the sham (3 mice), I45minR24h (4 mice), and I1hR24h (4 mice) group were isolated by centrifugation (2000 × g, 4°C, 10 min). Aspartate transaminase (AST), lactate dehydrogenase (LDH), creatine kinase (CK), and creatine kinase isoenzyme (CK‐MB) levels were measured using Beckmann AU680 (Beckman Coulter, Inc.) according to the manufacturer's instructions.

### 2.4 Cardiac EV isolation

EV isolation was performed according to our previous study.^10^ Briefly, the mice were sacrificed by cervical dislocation. After phosphate‐buffered saline (PBS) perfusion, left ventricle tissues subjected to IR injury were removed and cut into small pieces. The heart tissues were incubated in 4 ml of 0.1% type II collagenase (Sigma) at 37°C for 30 min. The digested tissue was centrifuged at 300 × g for 5 min at 4°C to remove the tissues and cells. The supernatant was centrifuged at 2000 × g for 10 min, and then at 10000 × g for 10 min at 4°C. The supernatant was centrifuged at 120,000 × g for 2 h at 4°C (Optima L‐100XP Ultracentrifuge, Beckman Coulter). Then, cardiac EVs were obtained after one wash with PBS.

### 2.5 EV quantification

The particle size and concentration of the EVs were tested using the ZetaView® NTA technique by Particle Metrix with 3 times replicates.

### 2.6 Transmission electron microscope (TEM)

The fresh‐isolated EVs were fixed with 2.5% glutaraldehyde and loaded onto 200 Mesh carbon‐coated formvar grids for 5 min. Then 2% phosphotungstic acid was used for EV‐staining (5 min at room temperature). EVs were detected under the transmission electron microscope (TEM; Hitachi, HT7700).

### 2.7 Western blot

EVs from different groups were lysed with RIPA buffer. Protein quantification was carried out using Bicinchoninic acid assay (BCA) protein estimation kit (Thermofisher Scientific) according to the manufacturer's instruction. Proteins were extracted and loaded on 10% SDS‐PAGE gels, then transferred to PVDF membranes. After blocking with 3% non‐fat milk in Tris‐buffered saline with 0.1% Tween 20 (TBST) for 1 h at room temperature, the membranes were incubated overnight at 4°C with primary antibodies directed to: CD9 (Abcam ab92726, 1:1000 dilution), Alix (Abcam ab186429, 1:1000 dilution), TSG101 (Abcam ab125011, 1:000 dilution). After TBST washing, the membranes were incubated with corresponding secondary antibodies (CST 7076 S, 1:1000 dilution) for 1 h. Then, specific bands were detected by ECL reagent (Share‐Bio).

### 2.8 RNA library construction and RNA sequencing

Cardiac EVs from 4 sham and 4 I45minR24h mice were isolated for RNA sequencing. Total RNA was isolated using TRIzol reagent (Invitrogen). The concentration and purity of RNAs were determined by a NanoDrop® ND‐2000 spectrophotometer (Thermo). After rRNA depletion with the Ribo‐Zero Magnetic Gold Kit (Epicentre, Inc), the RNA libraries were constructed using the TruSeq Stranded Total RNA Library Prep Kit (Illumina) according to the manufacturer's instructions. The BioAnalyzer 2100 system (Agilent Technologies, Inc) was used for library quality assessment. The RNA libraries were denatured as single‐strand DNA, captured on Illumina Flow Cells (Illumina), amplified in situ as clusters, and sequenced using a HiSeq 4000 sequencing system (Illumina). The reads of the lncRNAs are presented in Table S1.

### 2.9 RNA sequencing analysis

Paired‐end reads were obtained from an Illumina HiSeq 4000 sequencer. After 3′ adaptor‐trimming and low‐quality read removal by cutadapt software (v1.9.3), the high‐quality reads were aligned to the mouse reference genome (UCSC mm10) with Hisat2 software. The Cuffdiff software was used to calculate the fragments per kilobase per million (FPKM) for the expression profiles of lncRNAs and mRNAs. Differentially expressed (DE) genes were identified according to the criteria of fold change >2 and adjusted *p*‐Value <0.05. RNA‐seq data have been deposited in the GEO database under accession numbers GSE189888. LncRNA target genes were predicted based on cis functional prediction. Gene Ontology (GO) and pathway analysis were performed for these target genes.

### 2.10 LncRNA‐miRNA‐mRNA ceRNA network construction

Pathway analysis found several DE genes which were enriched in the T cell receptor signalling pathway and cell adhesion molecules (CAMs). The miRNAs interacting with the DE‐mRNAs were predicted by the TargetScan and Miranda databases. The top 5 predicted miRNAs of each gene were selected. Then, the selected miRNAs were used to find their target DE‐lncRNAs using the TargetScan and Miranda databases, and the top 5 target lncRNAs were selected. The miRNA‐mRNA and lncRNA‐miRNA pairs were used to build the lncRNA‐miRNA‐mRNA ceRNA network.

### 2.11 Quantitative real‐time polymerase chain reaction analysis

Real‐time quantitative polymerase chain reaction (RT‐qPCR) was used to verify the accuracy of the RNA‐seq data with 3 replicates. Total RNA was isolated using TRIzol reagent (Invitrogen) and reverse transcribed to cDNA using the PrimeScript RT reagent kit (TaKaRa Bio, Inc). The RT‐qPCR was performed on the Applied Biosystems ViiA 7 Real‐Time PCR System (Thermo Fisher Scientific) with SYBR Green MasterMix (Applied Biosystems; Thermo Fisher Scientific, Inc). The relative expression levels of lncRNAs and mRNAs were calculated using the 2^−∆∆CT^ method. The primers used in the study are listed in Table S2.

### 2.12 Statistical Analysis

The data were presented as mean ± standard error of mean (SEM). The Student's *t*‐test was applied for the comparison of the two groups. Multiple groups comparison was performed using one‐way analysis of variance (anova), followed by Tukey's multiple comparisons test. Correlation analysis was performed using Pearson's linear correlation analysis. GraphPad Prism 8.0 (GraphPad Software) was used for all data analyses. A *p*‐Value <0.05 was considered statistically significant.

## 3 RESULTS

### 3.1 The feature of cardiac EVs

**FIGURE 1:**
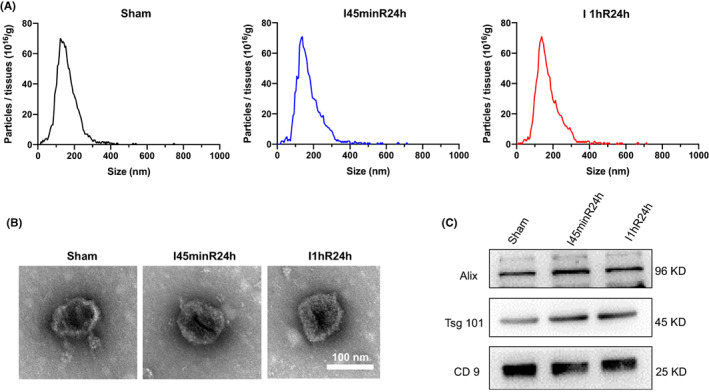
Characterization of cardiac EVs. (A) Particle size distribution of the EVs was measured using nanoparticle tracking analysis (NTA). (B) A representative TEM image of the EVs (bar = 100 nm). (C) Western blot showing the typical exosomal markers (Alix, Tsg101, and CD9) in the EVs.

The size distribution of EVs derived from the heart of the sham, I45minR24h, and I1hR24h mice was determined by NTA (Figure 1A). The typical morphology of EVs was captured under TEM (Figure 1B). The exosome markers including Alix, TSG101, and CD9 were expressed in these EVs from different conditions (Figure 1C).

### 3.2 Positive correlation between the severity of IR injury and the number of cardiac EVs

**FIGURE 2:**
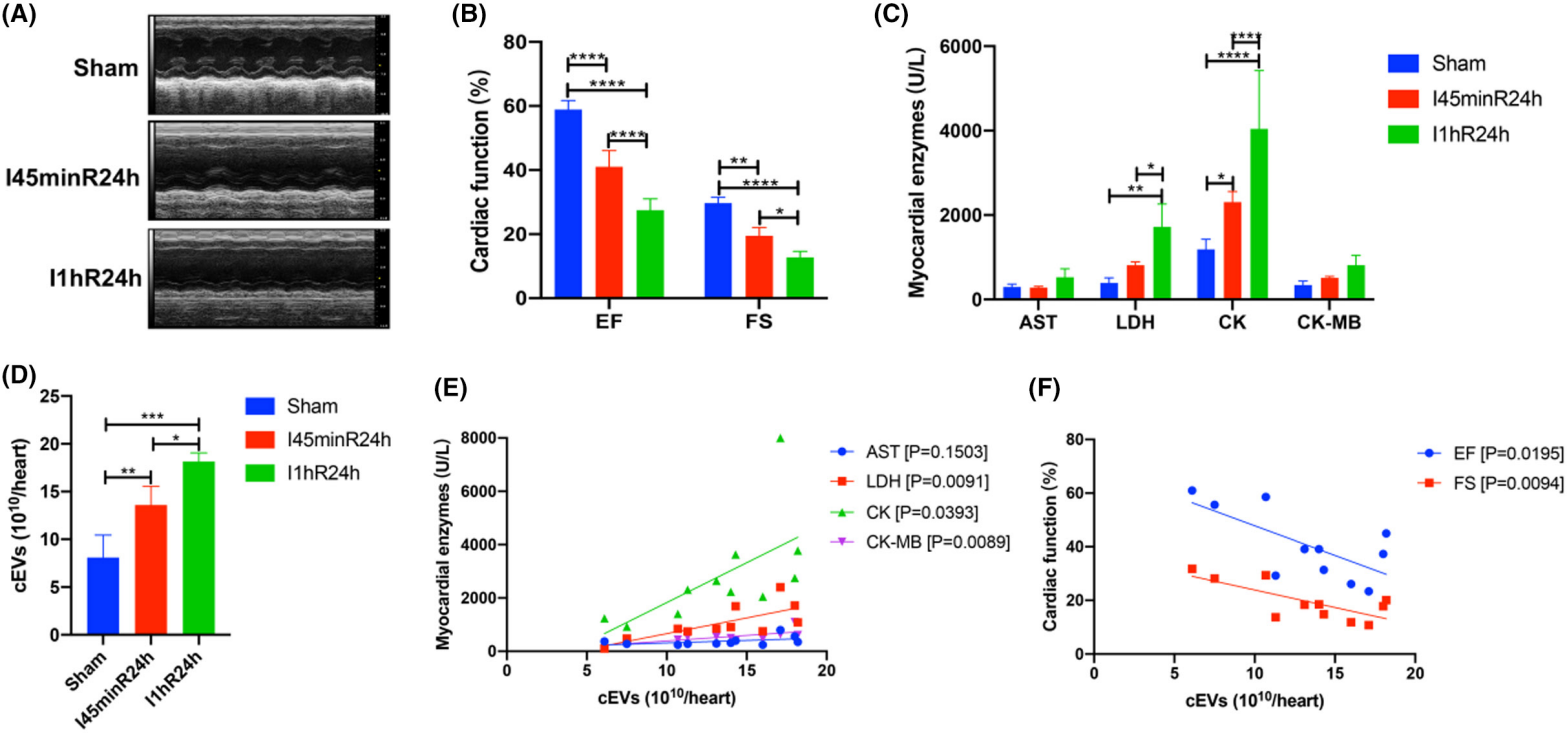
The association between the severity of IR injury and the number of cardiac EVs. (A) Representative echocardiography images of the sham, I45minR24h, and I1hR24h mice. (B) Longer‐term ischemia resulted in decreased cardiac function as determined by EF and FS. (C) Longer‐term ischemia led to increased release of myocardial enzymes (including AST, LDH, CK, and CK‐MB). (D) Longer‐term ischemia before reperfusion resulted in increased EV production. (E) The correlation between the number of EVs and the release of myocardial enzyme. (F) The correlation between the number of EVs and cardiac function. *p < 0.05; **p < 0.01; ***p < 0.001; ****p < 0.0001.

We recently found that myocardial IR significantly increased the intra‐cardiac release of EVs.^10^ To estimate the potential correlation between the severity of IR injury and cardiac EV production, we established myocardial IR models with different ischemia times (0 min, 45 min, 1 h). Longer‐term ischemia induced more severe myocardial dysfunction and IR injury as determined by echocardiography (Figures 2A,B) and myocardial enzyme (including AST, LDH, CK, and CK‐MB) analysis (Figure 2C). Meanwhile, longer‐term ischemia before reperfusion resulted in increased EV production (Figure 2D). In addition, significant linear correlations were found between the release of EVs and the serum levels of LDH (R^2^ = 0.5488; *p* = 0.0091), CK (R^2^ = 0.3921; *p* = 0.0393), and CK‐MB (R^2^ = 0.5506; *p* = 0.0089) (Figure 2E). Significant linear correlations were also demonstrated between the number of EVs and cardiac function, including the ejection fraction (EF, R^2^ = 0.4720; *p* = 0.0195) and fractional shortening (FS, R^2^ = 0.5458; *p* = 0.0094) (Figure 2F). These results suggested a close association between the severity of the heart injury and the increase in EV production post‐IR.

### 3.3 Differential lncRNA and mRNA profile in cardiac EVs after IR injury

**FIGURE 3:**
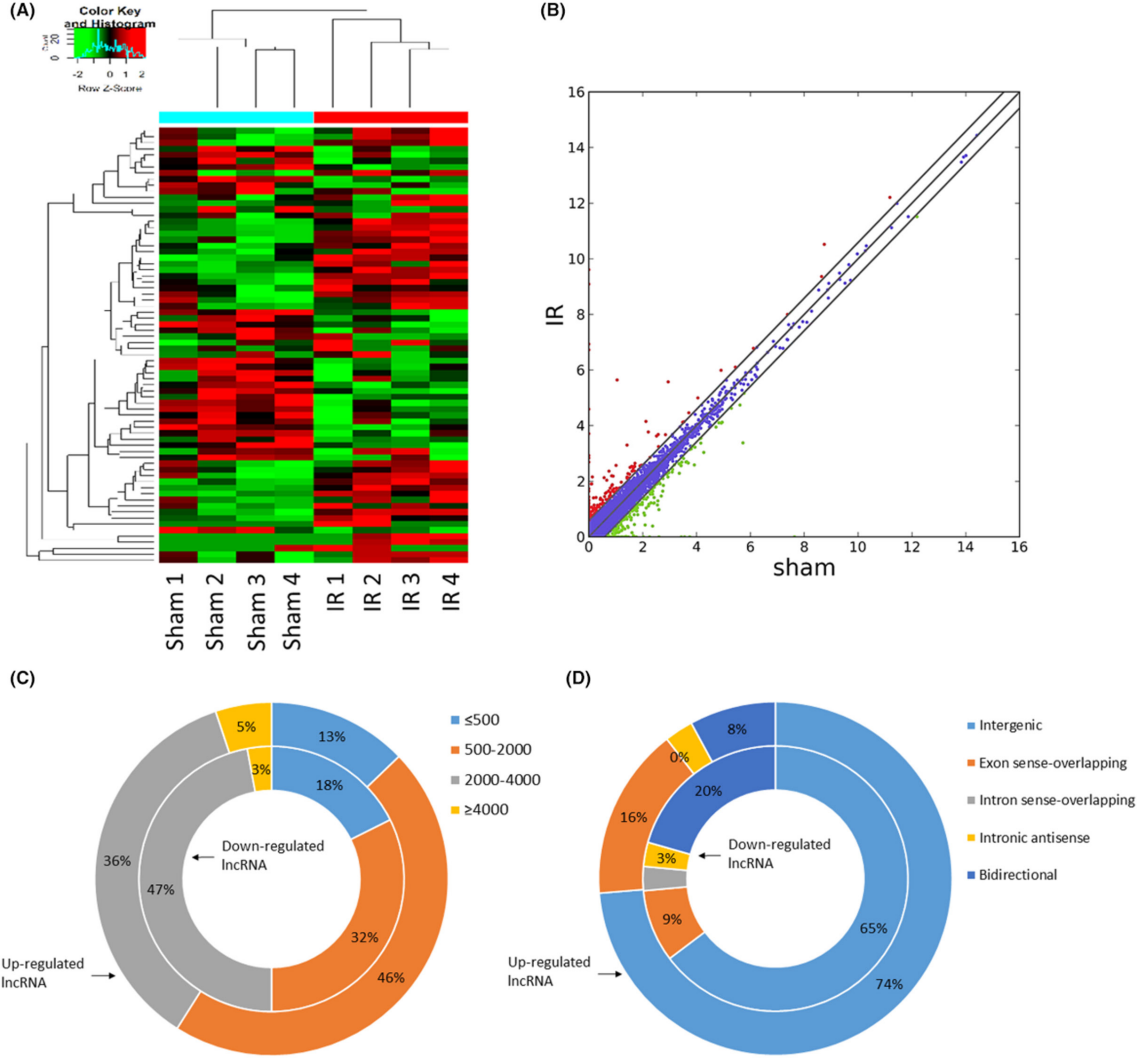
Differential expression of lncRNAs in EVs between the sham and IR groups. (A) Clustered heat map of the DE‐lncRNAs. (B) Scatter plot of the DE‐lncRNAs. Significantly up‐and downregulated lncRNAs are shown as red and green dots, respectively. (C) The distribution of DE‐lncRNAs based on the length of nuclear acids. (D) The distribution of DE‐lncRNAs based on their categories. The inner rings in (C, D) exhibit downregulated lncRNAs, and the outer rings in (C, D) exhibit upregulated lncRNAs.

Next, we detected the alteration of lncRNAs in the EVs from mice post‐myocardial IR injury and the mice with the sham operation. A total of 73 significantly differentially expressed lncRNAs, including 34 downregulated and 39 upregulated lncRNAs, were identified in the IR group (IR‐EVs) compared with the sham group (S‐EVs). The heat map of the DE‐lncRNAs is presented in Figure 3A. Significantly up‐ and downregulated lncRNAs were shown as red and green dots on the scatter plot (Figure 3B). The top 10 DE‐lncRNAs are listed in Table S3. Among these DE‐lncRNAs, the upregulated lncRNAs were predominantly between 500 and 2000 nucleotides (nt) in length, and the downregulated lncRNAs were mainly between 2000 and 4000 nt in length (Figure 3C). Most of the DE‐lncRNAs were identified as intergenic lncRNAs (Figure 3D). The potential target genes of these DE‐lncRNAs are predicted according to the lncRNA cis‐regulatory mechanism and are presented in Table S4.

**FIGURE 4:**
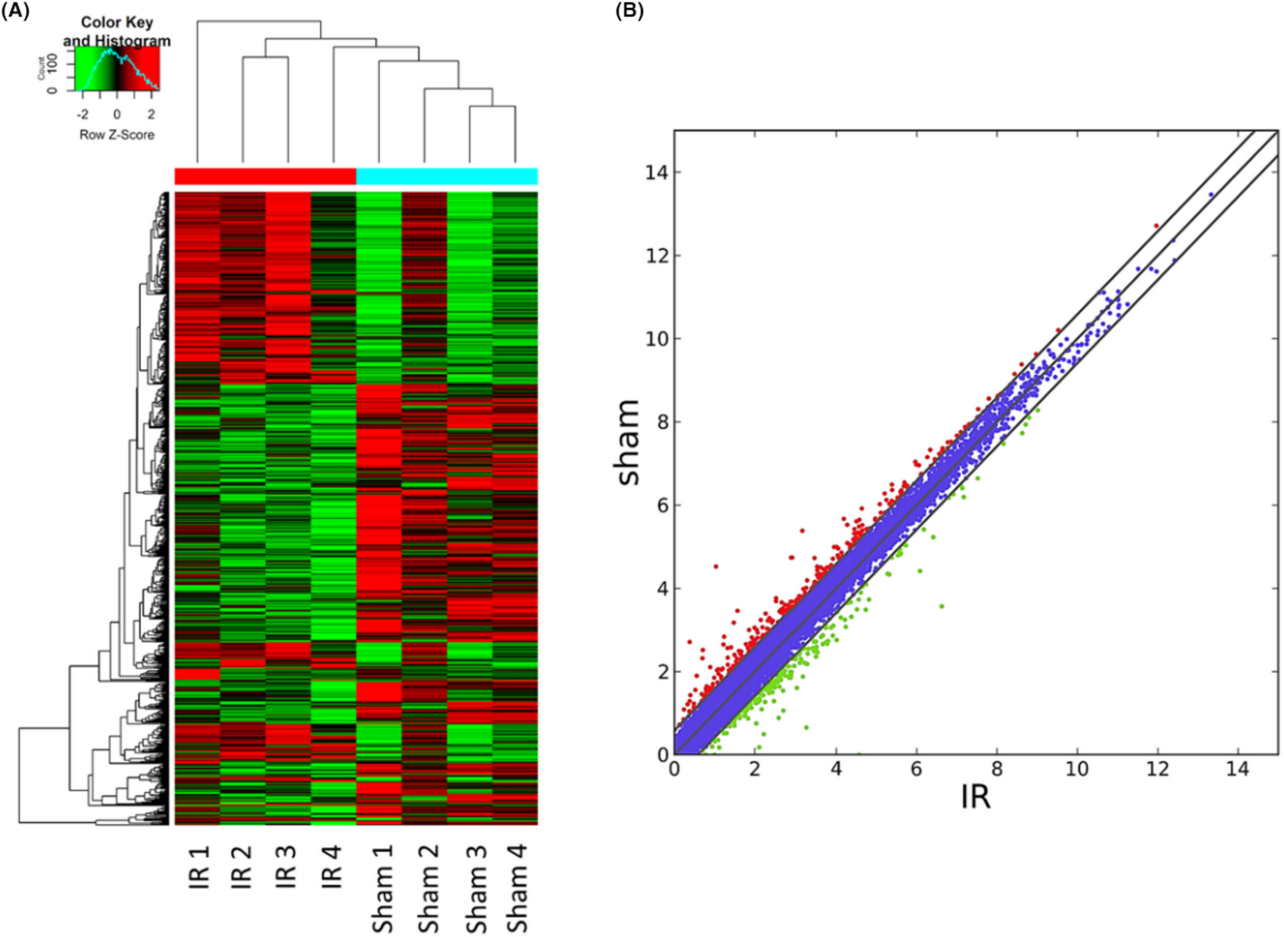
Differential expression of mRNAs in EVs between the sham and IR groups. (A) Clustered heat map of the DE‐mRNAs. (B) Scatter plot of the DE‐mRNAs. Significantly up‐ and downregulated mRNAs were shown as red and green dots, respectively.

In addition to the lncRNA expression profile, we also explored the mRNA expressions in these EVs. A total of 721 significant DE‐mRNAs were identified in the present study. Among these DE‐mRNAs, 404 mRNAs were downregulated and 317 mRNAs were upregulated in the IR‐EVs compared with the S‐EVs. The heat map showed the mRNA expressions between IR‐EVs and S‐EVs (Figure 4A). The scatter plot was presented comparing the two groups (Figure 4B). The top 10 DE‐mRNAs are listed in Table S5.

### 3.4 Validation of the RNA sequencing data

To confirm the differential expression of lncRNAs and mRNAs in the RNA‐seq data, we selected 4 DE‐lncRNAs (2 upregulated: AK138493 and ENSMUST00000098305) and 2 downregulated: (NR_045042 and NR_027923: Figures S1 A–D) and 4 DE‐mRNAs (2 upregulated: HMOX1 and Alox5 and 2 downregulated: SOCS3 and NOS2) (Figures S1 E–H). RT‐qPCR confirmed the significant differences in the expressions of all the selected RNAs between the two groups, which were consistent with the sequencing results.

### 3.5 GO analysis of the differentially expressed lncRNAs and mRNAs

**FIGURE 5:**
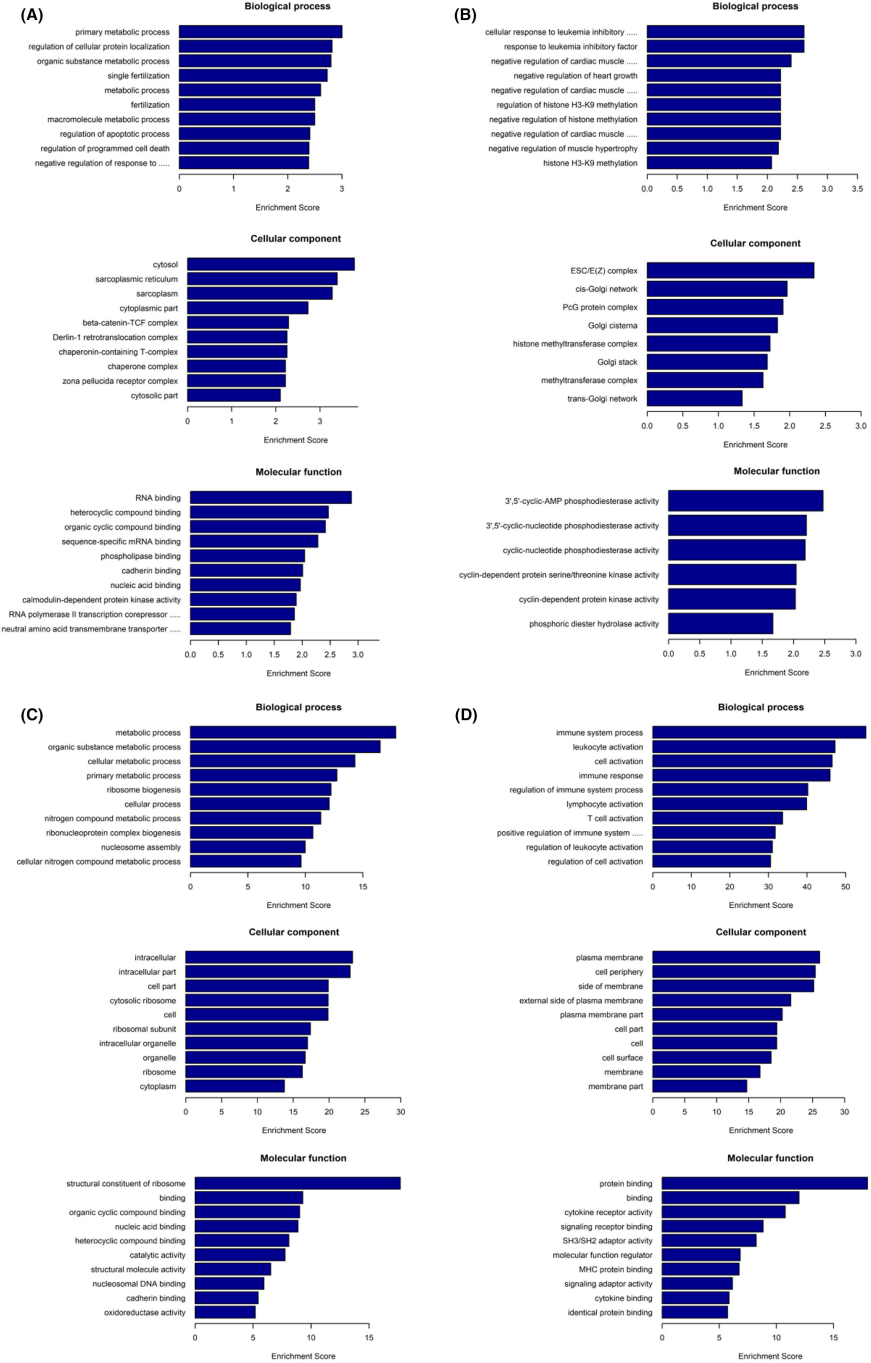
Predicted functions of the differentially expressed lncRNAs and mRNAs. GO analysis including biological process, cell component, and molecular function of (A) the downregulated lncRNAs, (B) the upregulated lncRNAs, (C) the downregulated mRNAs, and (D) the upregulated mRNAs.

The potential functions of the downregulated lncRNAs (Figure 5A) and upregulated lncRNAs (Figure 5B) were performed based on GO analysis of the target genes. GO analysis of the biological process suggested that the downregulated lncRNAs were primarily associated with the metabolic process (Figure 5A). The upregulated lncRNAs were largely involved in the response to leukaemia inhibitory factors (Figure 5B). GO cell component terms of the down‐ and upregulated lncRNAs were significantly enriched in the cytosol and ESC/E(Z) complex, respectively (Figures 5A,B). Molecular function GO analysis indicated that the down‐ and upregulated lncRNAs were mostly associated with RNA binding and phosphodiesterase activity, respectively (Figures 5A,B).

From the GO enrichment results of DE‐mRNAs in Figures 5C,D, we found that the downregulated mRNAs were mainly related to the metabolic process (Figure 5C). The upregulated mRNAs were generally involved in the immune system process (Figure 5D). The major GO cell component terms of the down‐ and upregulated mRNAs were intracellular part and plasma membrane, respectively (Figures 5C,D). GO analysis of molecular function suggested that the down‐ and upregulated lncRNAs were chiefly associated with the structural constituent of ribosome and protein binding, respectively (Figures 5C,D).

### 3.6 KEGG pathway analysis of the differentially expressed lncRNAs and mRNAs

**FIGURE 6:**
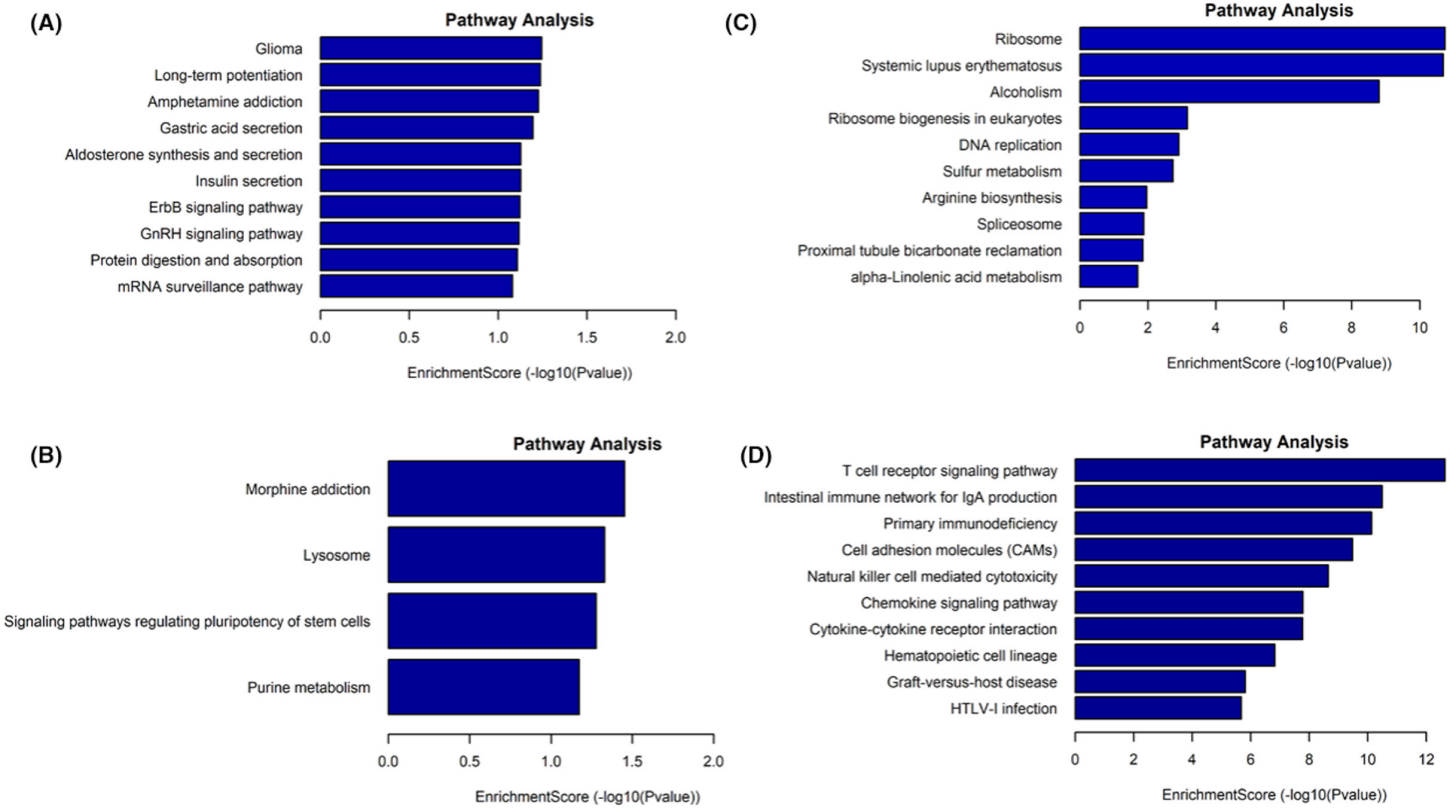
Predicted pathways of the differentially expressed lncRNAs and mRNAs. (A) Pathway analysis of the downregulated lncRNAs. (B) Pathway analysis of the upregulated lncRNAs. (C) Pathway analysis of the downregulated mRNAs. (D) Pathway analysis of the upregulated mRNAs.

Pathway analysis of the differentially expressed lncRNAs and mRNAs is presented in Figure 6. The top 10 of the significantly downregulated pathways of lncRNAs are shown in Figure 6A. The top 3 pathways were glioma, long‐term potentiation, and amphetamine addiction. The significantly upregulated pathways were morphine addiction, lysosome, signalling pathways regulating pluripotency of stem cells, and purine metabolism (Figure 6B). Regarding the pathway analysis of DE‐mRNAs, the top 10 significantly downregulated and upregulated pathways are shown in Figures 6C,D. The top 3 significantly downregulated pathways were ribosome, systemic lupus erythematosus, and alcoholism (Figure 6C). The top 3 significantly upregulated pathways included the T cell receptor signalling pathway, the intestinal immune network for IgA production, and primary immunodeficiency (Figure 6D).

### 3.7 The construction of lncRNA‐miRNA‐mRNA ceRNA network

**FIGURE 7:**
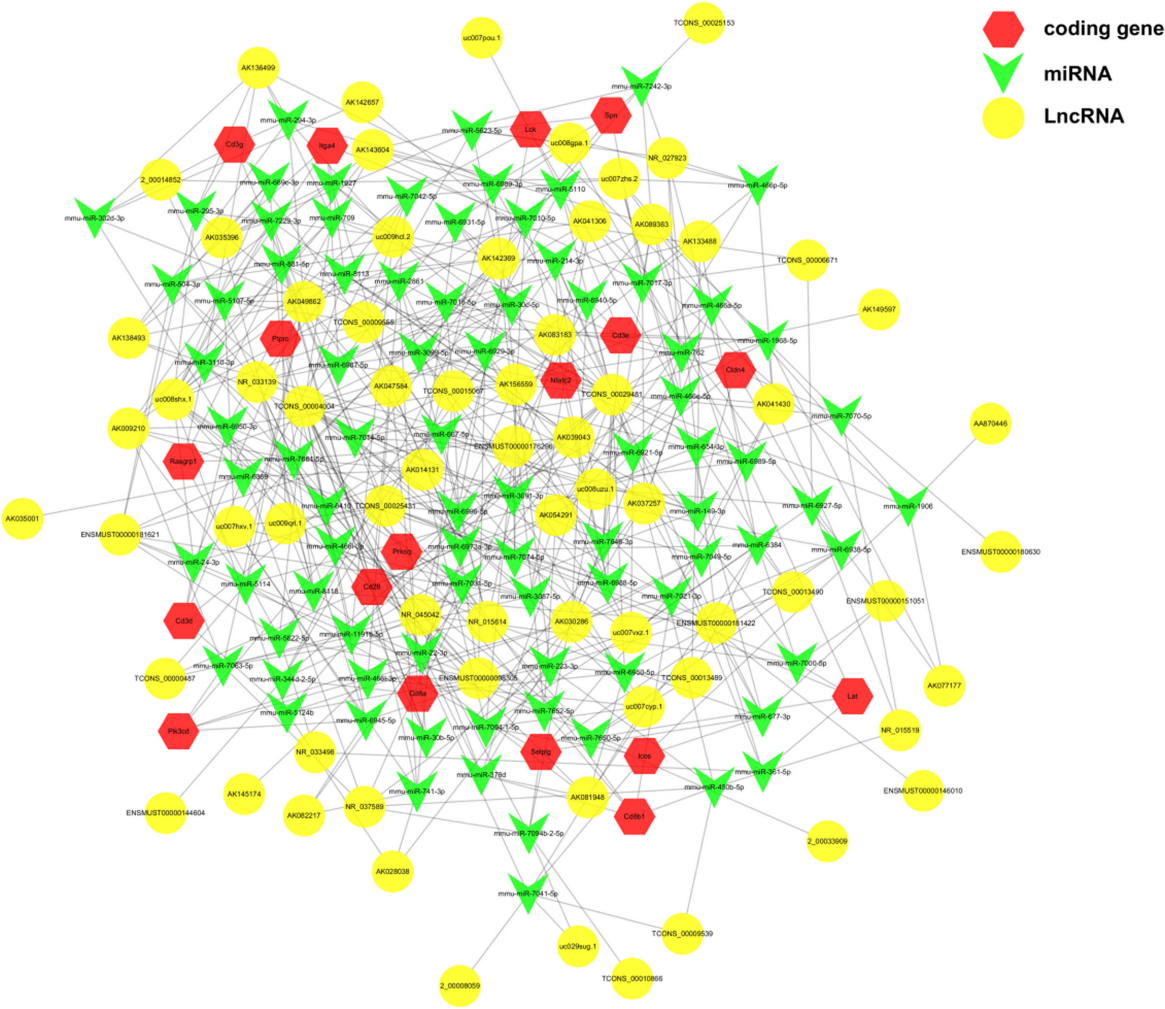
LncRNA‐miRNA‐mRNA ceRNA network based on the sequencing results. Each green arrow represents a miRNA which was core in the figure. Each yellow hexagon represents a lncRNA and each red hexagon represents an mRNA.

LncRNA is known to play important roles in the development of various diseases as a kind of ceRNA. Given the significant potential of IR‐EV contents on the immune regulation pathways especially the T cell receptor signalling pathway as shown in Figure 6, we constructed a lncRNA‐miRNA‐mRNA ceRNA network (Figure 7). Based on the DE genes (red hexagon in Figure 7) enriched in the T cell receptor signalling pathway and cell adhesion molecules, we obtained 90 mRNA‐miRNA pairs and 413 lncRNA‐miRNA pairs through binding sites analysis predicted by the TargetScan and Miranda databases. In the selected lncRNAs, there are 37 upregulated and 31 downregulated lncRNAs in IR‐EVs. The lncRNA‐miRNA‐mRNA ceRNA network suggested the possible functions of DE‐lncRNAs with mRNAs in the T cell receptor signalling pathway and cell adhesion molecules in the pathology of cardiac IR injury.

### 3.8 The correlation between the severity of IR injury and the DE‐lncRNAs

**FIGURE 8:**
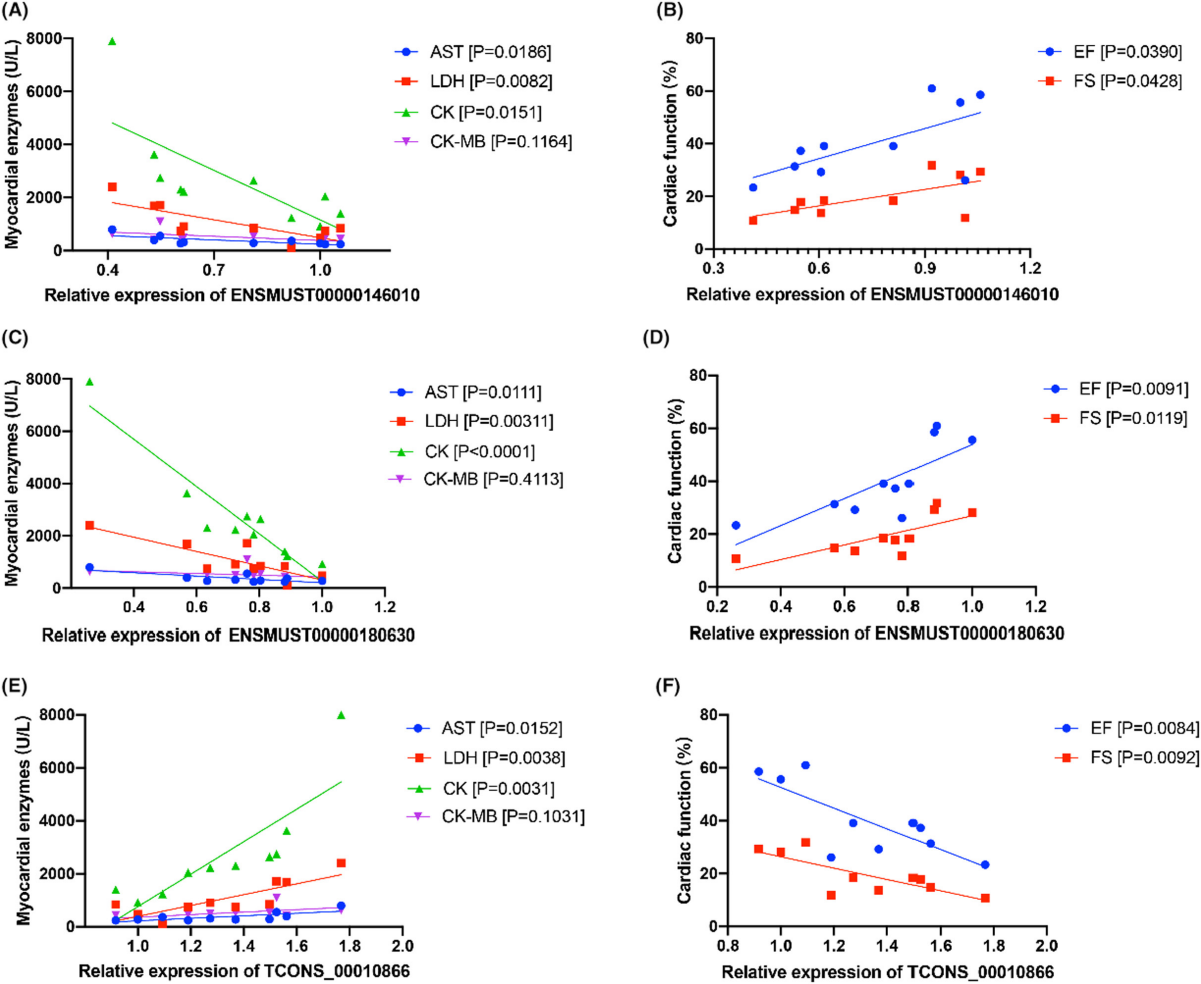
The correlation between the severity of IR injury and the DE‐lncRNAs. Linear correlations between the expression of ENSMUST00000146010 and (A) the release of myocardial enzymes and (B) cardiac function. Linear correlations between the expression of ENSMUST00000180630 and (C) the release of myocardial enzymes and (D) cardiac function. Linear correlations between the expression of TCONS_00010866 and (E) the release of myocardial enzymes and (F) cardiac function.

Next, to preliminarily evaluate the role of the DE‐lncRNAs in heart injury, we selected 5 DE‐lncRNAs from the top 10 highly expressed ones for further correlation analysis (Figure 8 and Table S6). Significant negative linear correlations were found between the expression of ENSMUST00000146010 and the levels of AST (R^2^ = 0.5198; *p* = 0.0186), LDH (R^2^ = 0.6037; *p* = 0.0082) and CK (R^2^ = 0.5429; *p* = 0.0151) (Figure 8A). A positive correlation between ENSMUST00000146010 expression and cardiac function determined by EF and FS is also shown in Figure 8B. ENSMUST00000180630 expression showed the similar correlation with these indexes including AST (R^2^ = 0.5742; *p* = 0.0111), LDH (R^2^ = 0.6854; *p* = 0.0031), CK (R^2^ = 0.8881; *p* < 0.0001), EF (R^2^ = 0.5932; *p* = 0.0091) and FS (R^2^ = 0.5676; *p* = 0.0119) (Figures 8C,D). In contrast, we found significant positive linear correlations between the expression of TCONS_00010866 and the severity of cardiac injury determined by AST (R^2^ = 0.5418; *p* = 0.0152), LDH (R^2^ = 0.6693; *p* = 0.0038), CK (R^2^ = 0.6861; *p* = 0.0031), EF (R^2^ = 0.6018; *p* = 0.0084) and FS (R^2^ = 0.5923; *p* = 0.0092) (Figures 8E,F). The statistical values of R^2^, *p* Values, and equations involved in the correlation analysis are concluded in Table S6.

## 4 DISCUSSION

Increasing evidence demonstrated the essential roles of EVs in the diagnosis, treatment, and pathological mechanism of cardiovascular diseases.^22^, ^23^ Little is known about the content and function of EVs from IR‐injured hearts. The present study identified the specific profile of lncRNAs and mRNAs in EVs derived from IR‐injured hearts and portrayed their potential functions and pathways. Interestingly, we found that the severity of IR injury was significantly correlated with the EV production in the injured heart and further identified significant correlations between the expression of 3 DE‐lncRNAs and the severity of IR injury. These results indicated the potential fundamental roles of the IR‐EVs and their lncRNAs cargo in the pathologic process of IR injury.

To determine the role of EVs in the pathogenesis and development of ischemic heart diseases, previous studies largely focused on the function of EVs/exosomes from mouse/ human blood. Myocardial infarction‐induced EVs were reported to allow a systemic response to cardiac injury by mobilizing bone marrow progenitor cells.^24^ Circulating EVs are derived from different organs/tissues in our body. Recent study revealed that intra‐cardiac EVs can trigger the inflammatory responses in monocyte in vitro.^25^ Our previous study found that IR increased the EV release in murine hearts.^10^ In addition, these IR‐EVs contribute to local and systemic inflammation.^11^ In the present study，we obtained EVs from IR‐injured heart and further revealed a significant correlation between EV quantity and the severity of IR injury, suggesting that cardiac EVs enriched lncRNA and mRNA may represent important players which contribute to cardiac IR injury.

A recent study reported that lncRNAs were actively secreted into the circulation during cardiac remodelling.^26^ Regulation of unique lncRNA molecules can attenuate hypoxia‐induced injury in cardiomyocytes,^27^ attenuate cardiac fibrosis^28^, ^29^ and regulate cardiomyocyte proliferation and cardiac repair.^30^ Therefore, lncRNAs are considered to serve as novel clinical biomarkers and targets for therapeutic drug development. Further studies highlighted the role of EVs as RNAs or protein vehicles mediating the initiation and progression of cardiac diseases. LncRNA‐enriched vesicles produced by hypoxic cardiomyocytes trigger cardiac fibrosis.^31^ In addition, atorvastatin enhanced the therapeutic efficacy of mesenchymal stem cell‐derived exosomes in acute myocardial infarction by upregulating LncRNA H19.^32^ In the present study, we identified a total of 73 DE‐lncRNAs and 721 DE‐mRNAs in EVs post‐IR injury. GO analysis of biological processes suggested the involvement of significantly altered RNAs in the metabolic processes and immune system processes. Cell metabolism and the immune response are vital processes in IR injury. Myocardial IR can cause enhanced fatty acid oxidation, impaired pyruvate oxidation, and accelerated anaerobic glycolysis.^33^, ^34^ These metabolic alterations directly influence tissue inflammation, integrity, and cell survival. In addition, targeting metabolic events is reported to be a promising strategy to reduce IR injury.^35^ We recently reported the role of IR‐EVs and their cargo miR155‐5p in macrophages polarization and cardiac injury post‐IR.^11^ The present results suggested that IR induced cardiac EVs may also regulate the metabolic and inflammatory responses in the injured heart through the differentially expressed lncRNAs.

KEGG pathway analysis of the DE‐lncRNAs/mRNAs revealed some important signalling pathways, which helped to elucidate the possible mechanisms. For example, in the downregulated lncRNA‐predicted pathways, neuregulin/ErbB signalling is essential for normal cardiac development and participants in hypertrophic growth and survival of embryonic, postnatal, and adult ventricular myocytes.^36^, ^37^, ^38^ Recent studies confirmed that hypoxia‐inducible factor 2a induced high expression of ErbB1 protected the heart from IR injury.^39^ Remote ischemic preconditioning can protect the heart against IR injury through activation of NRG‐1/ErbB3 signalling.^40^ In the downregulated lncRNA predicted pathways, purine metabolism signalling plays an important role in cardiovascular diseases. Purines participate in heart activities and vagal cardiovascular reflexes and act on purinoceptors of different kinds of cells.^41^ Moreover, a clinical study identified the association between right ventricular‐pulmonary vascular dysfunction and purine metabolites in pulmonary hypertension patients.^42^ The DE‐mRNA predicted pathways (e.g., T cell receptor signalling pathway, cell adhesion molecules, and chemokine signalling pathway) are known to participate in heart injury and repair. T cells play crucial roles in heart repair and function during cardiac injury.^43^ CD4^+^ T cells produce various cytokines/chemokines that participate in the immune response and cardiac remodelling post‐IR injury.^44^, ^45^ Various chemokines are upregulated after IR as a result of cardiac damage and immune activation. Data from animal experiments underlined a cardioprotective effect of monocyte chemoattractant protein‐1/chemokine (C‐C motif) ligand 2 (MCP‐1/CCL2) inhibitor treatment on IR‐injured mice.^46^ MCP‐1 knockout protected the mice from adverse myocardial infarction remodelling.^47^ Therefore, IR‐EVs may participate in IR injury by transferring their mRNA cargo and regulating these pathways in target cells. Further studies are still needed to explore the specific mechanism of these DE‐lncRNAs/mRNAs in IR injury.

It has been demonstrated that miRNA‐mediated crosstalk between mRNAs and lncRNAs is well‐organized as a lncRNA‐miRNA‐mRNA ceRNA network in various biological processes. LncRNAs, serving as miRNA sponges, play important roles in heart diseases.^48^ Previous studies confirmed the vital roles of T cells in heart injury and repair.^49^ Given the significant potential of EV cargos on the immune regulation pathway especially the T cell receptor signalling pathway and cell adhesion molecules, we constructed a lncRNA‐miRNA‐mRNA ceRNA network to elucidate the role of cardiac EV‐delivered lncRNAs on T cells regulation and cell adhesion. Although further explorations were needed to confirm these functions in vivo, the lncRNA‐miRNA‐mRNA network provides the possible novel targets for the mechanism investigation of cardiac IR injury.

Previous studies have confirmed the use of exosomal lncRNAs as biomarkers for cancers.^50^, ^51^ In the present study, we validated several DE‐lncRNAs in IR‐EVs and found their correlation with the severity of IR injury. The significant correlations indicated the importance of these DE‐lncRNAs in IR injury and the potential of the IR‐EVs content and DE‐lncRNAs and mRNA in EVs as biomarkers to predict IR injury. Further studies are warranted to explore the specific mechanism and disclose how these DE‐lncRNAs and mRNA are involved in cardiac IR injury. For translational application, further explorations to confirm the expressions of the differential expressed lncRNAs in EVs derived from peripheral blood and their associations with the severity of IR injury in patient cohorts are needed. Besides, cardiac EVs are released by different cell types, such as cardiomyocytes, endothelial cells, and fibroblasts. Our previous study found an increased release of cardiomyocyte‐derived (CX43^+^) and endothelium‐derived (CD106^+^) EVs in the IR‐injured heart. However, how EVs with different origins exert their functions during IR injury need further exploration.

## 5 CONCLUSION

The present study for the first time identified the lncRNA and mRNA profiles of IR‐induced EVs in the heart and disclosed the correlations between the severity of IR injury and the number of EVs and the DE‐lncRNAs in IR‐EVs. GO and pathway analysis of the DE‐lncRNAs and DE‐mRNAs indicated diverse roles of cardiac EVs in the pathological process of IR injury. Therefore, IR‐EVs and the identified DE‐lncRNAs may contribute to cardiac injury and serve as novel biomarkers for injury assessment.

## AUTHOR CONTRIBUTIONS

**Xinyu Ge:** Data curation (lead); formal analysis (lead); investigation (lead); writing – original draft (lead). **Qingshu Meng:** Investigation (equal); methodology (equal); resources (equal). **Xuan Liu:** Investigation (equal); methodology (equal); resources (equal). **Jing Liu:** Investigation (equal); methodology (supporting). **Xiaoxue Ma:** Investigation (supporting); methodology (supporting); resources (supporting). **Shanshan Shi:** Investigation (supporting); methodology (supporting). **Mimi Li:** Investigation (supporting); methodology (supporting); resources (supporting). **Fang Lin:** Methodology (supporting); resources (supporting). **Xiaoting Liang:** Formal analysis (supporting); resources (supporting). **Xin Gong:** Investigation (supporting); methodology (supporting). **Zhong min Liu:** Project administration (supporting); resources (supporting). **Wei Han:** Supervision (equal). **Xiaohui Zhou:** Conceptualization (lead); funding acquisition (lead); project administration (lead); supervision (lead); writing – review and editing (lead).

## CONFLICT OF INTEREST

The authors have no conflict of interest.

## Supporting information


AppendixS1

## Data Availability

The datasets generated for this study can be found in the Gene expression Omnibus, accession GSE189888.

## References

[1] Yellon DM , Hausenloy DJ . Myocardial reperfusion injury. N Engl J Med. 2007;357(11):1121‐1135. 17855673 10.1056/NEJMra071667

[2] Nieuwland R , Falcon‐Perez JM , Soekmadji C , Boilard E , Carter D , Buzas EI . Essentials of extracellular vesicles: posters on basic and clinical aspects of extracellular vesicles. J Extracell Vesicles. 2018;7(1):1548234. 30533205 10.1080/20013078.2018.1548234 PMC6282440

[3] Tkach M , Thery C . Communication by extracellular vesicles: where we are and where we need to go. Cell. 2016;164(6):1226‐1232. 26967288 10.1016/j.cell.2016.01.043

[4] Cabral J , Ryan AE , Griffin MD , Ritter T . Extracellular vesicles as modulators of wound healing. Adv Drug Deliv Rev. 2018;129:394‐406. 29408181 10.1016/j.addr.2018.01.018

[5] Todorova D , Simoncini S , Lacroix R , Sabatier F , Dignat‐George F . Extracellular vesicles in angiogenesis. Circ Res. 2017;120(10):1658‐1673. 28495996 10.1161/CIRCRESAHA.117.309681 PMC5426696

[6] Maas SLN , Breakefield XO , Weaver AM . Extracellular vesicles: unique intercellular delivery vehicles. Trends Cell Biol. 2017;27(3):172‐188. 27979573 10.1016/j.tcb.2016.11.003 PMC5318253

[7] Colombo M , Raposo G , Thery C . Biogenesis, secretion, and intercellular interactions of exosomes and other extracellular vesicles. Annu Rev Cell Dev Biol. 2014;30:255‐289. 25288114 10.1146/annurev-cellbio-101512-122326

[8] Abels ER , Breakefield XO . Introduction to extracellular vesicles: biogenesis, RNA cargo selection, content, release, and uptake. Cell Mol Neurobiol. 2016;36(3):301‐312. 27053351 10.1007/s10571-016-0366-z PMC5546313

[9] Crewe C , Joffin N , Rutkowski JM , et al. An endothelial‐to‐adipocyte extracellular vesicle axis governed by metabolic state. Cell. 2018;175(3):695‐708.e13. 30293865 10.1016/j.cell.2018.09.005 PMC6195477

[10] Ge X , Meng Q , Zhuang R , et al. Circular RNA expression alterations in extracellular vesicles isolated from murine heart post ischemia/reperfusion injury. Int J Cardiol. 2019;296:136‐140. 31466885 10.1016/j.ijcard.2019.08.024

[11] Ge X , Meng Q , Wei L , et al. Myocardial ischemia‐reperfusion induced cardiac extracellular vesicles harbour proinflammatory features and aggravate heart injury. J Extracell Vesicles. 2021;10(4):e12072. 33664937 10.1002/jev2.12072 PMC7902529

[12] Mercer TR , Dinger ME , Mattick JS . Long non‐coding RNAs: insights into functions. Nat Rev Genet. 2009;10(3):155‐159. 19188922 10.1038/nrg2521

[13] Han P , Li W , Lin CH , et al. A long noncoding RNA protects the heart from pathological hypertrophy. Nature. 2014;514(7520):102‐106. 25119045 10.1038/nature13596 PMC4184960

[14] Huang Y . The novel regulatory role of lncRNA‐miRNA‐mRNA axis in cardiovascular diseases. J Cell Mol Med. 2018;22(12):5768‐5775. 30188595 10.1111/jcmm.13866 PMC6237607

[15] Piccoli MT , Gupta SK , Viereck J , et al. Inhibition of the cardiac fibroblast‐enriched lncRNA Meg3 prevents cardiac fibrosis and diastolic dysfunction. Circ Res. 2017;121(5):575‐583. 28630135 10.1161/CIRCRESAHA.117.310624

[16] Liu CY , Zhang YH , Li RB , et al. LncRNA CAIF inhibits autophagy and attenuates myocardial infarction by blocking p53‐mediated myocardin transcription. Nat Commun. 2018;9(1):29. 29295976 10.1038/s41467-017-02280-y PMC5750208

[17] Li Z , Zhang Y , Ding N , et al. Inhibition of lncRNA XIST improves myocardial I/R injury by targeting miR‐133a through inhibition of autophagy and regulation of SOCS2. Mol Ther Nucleic Acids. 2019;18:764‐773. 31734557 10.1016/j.omtn.2019.10.004 PMC6861669

[18] Shi H , Dong Z , Gao H . LncRNA TUG1 protects against cardiomyocyte ischaemia reperfusion injury by inhibiting HMGB1. Artif Cells Nanomed Biotechnol. 2019;47(1):3511‐3516. 31432688 10.1080/21691401.2018.1556214

[19] Su Q , Liu Y , Lv XW , et al. Inhibition of lncRNA TUG1 upregulates miR‐142‐3p to ameliorate myocardial injury during ischemia and reperfusion via targeting HMGB1‐ and Rac1‐induced autophagy. J Mol Cell Cardiol. 2019;133:12‐25. 31145943 10.1016/j.yjmcc.2019.05.021

[20] Lin F , Gong X , Yu P , et al. Distinct circulating expression profiles of long noncoding RNAs in heart failure patients with ischemic and nonischemic dilated cardiomyopathy. Front Genet. 2019;10:1116. 31781171 10.3389/fgene.2019.01116 PMC6861296

[21] Viereck J , Thum T . Circulating noncoding RNAs as biomarkers of cardiovascular disease and injury. Circ Res. 2017;120(2):381‐399. 28104771 10.1161/CIRCRESAHA.116.308434

[22] Bellin G , Gardin C , Ferroni L , et al. Exosome in cardiovascular diseases: a complex world full of hope. Cell. 2019;8(2):166. 10.3390/cells8020166 PMC6406975 30781555

[23] Sluijter JPG , Davidson SM , Boulanger CM , et al. Extracellular vesicles in diagnostics and therapy of the ischaemic heart: position paper from the working group on cellular biology of the heart of the European Society of Cardiology. Cardiovasc Res. 2018;114(1):19‐34. 29106545 10.1093/cvr/cvx211 PMC5852624

[24] Cheng M , Yang J , Zhao X , et al. Circulating myocardial microRNAs from infarcted hearts are carried in exosomes and mobilise bone marrow progenitor cells. Nat Commun. 2019;10(1):959. 30814518 10.1038/s41467-019-08895-7 PMC6393447

[25] Loyer X , Zlatanova I , Devue C , et al. Intra‐cardiac release of extracellular vesicles shapes inflammation following myocardial infarction. Circ Res. 2018;123(1):100‐106. 29592957 10.1161/CIRCRESAHA.117.311326 PMC6023578

[26] Kumarswamy R , Bauters C , Volkmann I , et al. Circulating long noncoding RNA, LIPCAR, predicts survival in patients with heart failure. Circ Res. 2014;114(10):1569‐1575. 24663402 10.1161/CIRCRESAHA.114.303915

[27] Huang Z , Ye B , Wang Z , et al. Inhibition of LncRNA‐HRIM increases cell viability by regulating autophagy levels during hypoxia/reoxygenation in myocytes. Cell Physiol Biochem. 2018;46(4):1341‐1351. 29689566 10.1159/000489149

[28] Zhang K , Zhang M , Yao Q , et al. The hepatocyte‐specifically expressed lnc‐HSER alleviates hepatic fibrosis by inhibiting hepatocyte apoptosis and epithelial‐mesenchymal transition. Theranostics. 2019;9(25):7566‐7582. 31695787 10.7150/thno.36942 PMC6831459

[29] Liang H , Pan Z , Zhao X , et al. LncRNA PFL contributes to cardiac fibrosis by acting as a competing endogenous RNA of let‐7d. Theranostics. 2018;8(4):1180‐1194. 29464008 10.7150/thno.20846 PMC5817119

[30] Ponnusamy M , Liu F , Zhang YH , et al. Long noncoding RNA CPR (Cardiomyocyte proliferation regulator) regulates Cardiomyocyte proliferation and cardiac repair. Circulation. 2019;139(23):2668‐2684. 30832495 10.1161/CIRCULATIONAHA.118.035832

[31] Kenneweg F , Bang C , Xiao K , et al. Long noncoding RNA‐enriched vesicles secreted by hypoxic Cardiomyocytes drive cardiac fibrosis. Mol Ther Nucleic Acids. 2019;18:363‐374. 31634682 10.1016/j.omtn.2019.09.003 PMC6807307

[32] Huang P , Wang L , Li Q , et al. Atorvastatin enhances the therapeutic efficacy of mesenchymal stem cells derived exosomes in acute myocardial infarction via up‐regulating long non‐coding RNA H19. Cardiovasc Res. 2019;16(2):353‐367. 10.1093/cvr/cvz139 PMC8204482 31119268

[33] Turer AT , Stevens RD , Bain JR , et al. Metabolomic profiling reveals distinct patterns of myocardial substrate use in humans with coronary artery disease or left ventricular dysfunction during surgical ischemia/reperfusion. Circulation. 2009;119(13):1736‐1746. 19307475 10.1161/CIRCULATIONAHA.108.816116 PMC2756963

[34] Frank A , Bonney M , Bonney S , Weitzel L , Koeppen M , Eckle T . Myocardial ischemia reperfusion injury: from basic science to clinical bedside. Semin Cardiothorac Vasc Anesth. 2012;16(3):123‐132. 22368166 10.1177/1089253211436350 PMC3457795

[35] Li T , Zhang Z , Kolwicz SC Jr , et al. Defective branched‐chain amino acid catabolism disrupts glucose metabolism and sensitizes the heart to ischemia‐reperfusion injury. Cell Metab. 2017;25(2):374‐385. 28178567 10.1016/j.cmet.2016.11.005 PMC5301464

[36] Zhao YY , Feron O , Dessy C , Han X , Marchionni MA , Kelly RA . Neuregulin signaling in the heart. dynamic targeting of erbB4 to caveolar microdomains in cardiac myocytes. Circ Res. 1999;84(12):1380‐1387. 10381889 10.1161/01.res.84.12.1380

[37] Nakaoka Y , Nishida K , Narimatsu M , et al. Gab family proteins are essential for postnatal maintenance of cardiac function via neuregulin‐1/ErbB signaling. J Clin Invest. 2007;117(7):1771‐1781. 17571162 10.1172/JCI30651 PMC1888569

[38] Honkoop H , de Bakker DE , Aharonov A , et al. Single‐cell analysis uncovers that metabolic reprogramming by ErbB2 signaling is essential for cardiomyocyte proliferation in the regenerating heart. Elife. 2019;8:e50163. 31868166 10.7554/eLife.50163 PMC7000220

[39] Lee JW , Koeppen M , Seo SW , et al. Transcription‐independent induction of ERBB1 through hypoxia‐inducible factor 2A provides cardioprotection during ischemia and reperfusion. Anesthesiology. 2019;132(4):763‐780. 10.1097/ALN.0000000000003037 PMC7072004 31794514

[40] Pilz PM , Hamza O , Gidlof O , et al. Remote ischemic perconditioning attenuates adverse cardiac remodeling and preserves left ventricular function in a rat model of reperfused myocardial infarction. Int J Cardiol. 2019;285:72‐79. 30904281 10.1016/j.ijcard.2019.03.003

[41] Burnstock G . Purinergic signaling in the cardiovascular system. Circ Res. 2017;120(1):207‐228. 28057794 10.1161/CIRCRESAHA.116.309726

[42] Lewis GD , Ngo D , Hemnes AR , et al. Metabolic profiling of right ventricular‐pulmonary vascular function reveals circulating biomarkers of pulmonary hypertension. J Am Coll Cardiol. 2016;67(2):174‐189. 26791065 10.1016/j.jacc.2015.10.072 PMC4962613

[43] Borg N , Alter C , Gorldt N , et al. CD73 on T cells orchestrates cardiac wound healing after myocardial infarction by purinergic metabolic reprogramming. Circulation. 2017;136(3):297‐313. 28432149 10.1161/CIRCULATIONAHA.116.023365

[44] Yuan D , Tie J , Xu Z , et al. Dynamic profile of CD4(+) T‐cell‐associated cytokines/chemokines following murine myocardial infarction/reperfusion. Mediators Inflamm. 2019;2019:9483647. 31011288 10.1155/2019/9483647 PMC6442492

[45] Hofmann U , Beyersdorf N , Weirather J , et al. Activation of CD4+ T lymphocytes improves wound healing and survival after experimental myocardial infarction in mice. Circulation. 2012;125(13):1652‐1663. 22388323 10.1161/CIRCULATIONAHA.111.044164

[46] Liehn EA , Piccinini AM , Koenen RR , et al. A new monocyte chemotactic protein‐1/chemokine CC motif ligand‐2 competitor limiting neointima formation and myocardial ischemia/reperfusion injury in mice. J Am Coll Cardiol. 2010;56(22):1847‐1857. 21087715 10.1016/j.jacc.2010.04.066

[47] Dewald O , Zymek P , Winkelmann K , et al. CCL2/monocyte chemoattractant Protein‐1 regulates inflammatory responses critical to healing myocardial infarcts. Circ Res. 2005;96(8):881‐889. 15774854 10.1161/01.RES.0000163017.13772.3a

[48] Tu S , Wang XY , Zeng LX , Shen ZJ , Zhang ZH . LncRNA TINCR improves cardiac hypertrophy by regulating the miR‐211‐3p‐VEGFB‐SDF‐1α‐CXCR4 pathway. Lab Invest. 2022;102(3):253‐262. 34732848 10.1038/s41374-021-00678-3

[49] Xia N , Lu Y , Gu M , et al. A unique population of regulatory T cells in heart potentiates cardiac protection from myocardial infarction. Circulation. 2020;142(20):1956‐1973. 32985264 10.1161/CIRCULATIONAHA.120.046789

[50] Zhan Y , Du L , Wang L , et al. Expression signatures of exosomal long non‐coding RNAs in urine serve as novel non‐invasive biomarkers for diagnosis and recurrence prediction of bladder cancer. Mol Cancer. 2018;17(1):142. 30268126 10.1186/s12943-018-0893-y PMC6162963

[51] Lin LY , Yang L , Zeng Q , et al. Tumor‐originated exosomal lncUEGC1 as a circulating biomarker for early‐stage gastric cancer. Mol Cancer. 2018;17(1):84. 29690888 10.1186/s12943-018-0834-9 PMC5978993

