# Supplementary material for: Alterations of long noncoding RNAs and mRNAs in extracellular vesicles derived from the murine heart post‐ischemia–reperfusion injury
Source: J Cell Mol Med. 2022 Nov 28;26(24):6006–18. doi: 10.1111/jcmm.17617 (PMC9753460; doi:10.1111/jcmm.17617)
Supplement: Supplementary file 1 — AppendixS1 [file JCMM-26-6006-s001.docx]

Supplementary material

Alterations of long noncoding RNAs and mRNAs in extracellular vesicles derived from the murine heart post ischemia-reperfusion injury

Xinyu Ge^1-3^, Qingshu Meng^1,2^, Xuan Liu^1-3^，Jing Liu^1-3^, Xiaoxue Ma^1,2^, Shanshan Shi^1,2^, Mimi Li^1,2^, Fang Lin^1-3^, Xiaoting Liang^4^, Xin Gong^5^，Zhongmin Liu^1-3,6^, Wei Han^5*^, Xiaohui Zhou^1,2*^

1. Research Center for Translational Medicine, Shanghai East Hospital, Tongji University School of Medicine, Shanghai 200120, P.R. China;
2. Shanghai Heart Failure Research Center, Shanghai East Hospital, Tongji University School of Medicine, Shanghai 200120, P.R. China;
3. Department of Cardiothoracic Surgery, Shanghai East Hospital, Tongji University School of Medicine, Shanghai 200120, P.R. China;
4. Institute for Regenerative Medicine, Shanghai East Hospital, School of Life Sciences and Technology, Tongji University, Shanghai 200120, P.R. China;
5. Department of Heart Failure, Shanghai East Hospital, Tongji University School of Medicine, Shanghai 200120, P.R. China;
6. Shanghai Institute of Stem Cell Research and Clinical Translation, Shanghai 200120, China.

Running title: LncRNA and mRNA profiles in cardiac EVs post IR injury

^*^ Address for Correspondence:

*Xiaohui Zhou, MD, PhD*

Research Center for Translational Medicine, Shanghai East Hospital, Tongji University School of Medicine, No. 150 Jimo Rd., Shanghai 200120, P.R. China. Tel: 0086-2161569884; Fax: 0086-2158798999; Email: zxh100@tongji.edu.cn or [xhzhou100@126.com](mailto:xhzhou100@126.com)

*Wei Han, MD*

Department of Heart failure, Shanghai East Hospital, School of Medicine, Tongji University, Shanghai, China 200120. Tel: 0086-38804518-18351; Fax: 0086-2158798999; E-mail address: dr.hanwei@foxmail.com

**Supplementary** **Figure 1**

**Supplementary Figure 1. Validation of the RNA-sequencing data. Relative expressions of 4 DE-lncRNAs and 4 DE-mRNAs in EVs were detected by qPCR.** The histograms of (A) NR_045042, (B) NR_027923, (C) AK138493, and (D) ENSMUST00000098305 are shown. Relative levels of 4 DE-mRNAs in EVs were detected by qPCR. The histograms of (E) SOCS3, (F) NOS2, (G) Hmox1, and (H) Alox5 are presented. *, P<0.05; **, P<0.01; ****, P<0.0001 compared to the sham group.

**Supplementary Table 1. The reads of lncRNAs.**

| **Sample** | **Raw reads** | **Clean reads** | **Aligned reads** | **Aligned rate** |
| --- | --- | --- | --- | --- |
| sham1 | 81,234,036 | 81,175,184 | 73,858,014 | 90.99% |
| sham2 | 82,709,744 | 82,645,582 | 72,251,601 | 87.42% |
| sham3 | 124,509,256 | 124,383,386 | 106,490,368 | 85.61% |
| sham4 | 110,790,448 | 110,719,326 | 93,650,353 | 84.58% |
| IR1 | 97,465,752 | 97,419,300 | 72,399,292 | 74.32% |
| IR2 | 100,528,424 | 100,442,132 | 84,860,122 | 84.49% |
| IR3 | 88,502,270 | 88,451,942 | 70,578,490 | 79.79% |
| IR4 | 75,136,388 | 75,042,242 | 68,637,409 | 91.47% |

**Supplementary Table 2. Quantitative polymerase chain reaction primers**

| **Gene** | **Primers** | |
| --- | --- | --- |
|  | **Forward primer (5'-3')** | **Reverse primer (5'-3')** |
| SOCS3 | ATGGTCACCCACAGCAAGTTT | TCCAGTAGAATCCGCTCTCCT |
| NOS2 | CACCAAGCTGAACTTGAGCGA | CCATAGGAAAAGACTGCACCGA |
| HMOX1 | ACAGAGGAACACAAAGACCAG | GTGTCTGGGATGAGCTAGTG |
| Alox5 | TGTCTGAGGTGTTTGGTATCG | AAGGCCATACTCGCAGATAAG |
| ENSMUST00000146010 | ACCCAAAGGTTGAGAGCTACTG | CAAGACCATCCCCTTCGTCC |
| TCONS_00010866 | AGCGAATGATTAGAGGTCTTGG | CCGTTCTGCTTACCAAAAGTG |
| NR_045042 | TGGGCGGGAATAAAGCGAAG | TTTAACCCACTCTGTGCTCCC |
| ENSMUST00000180630 | GGTCCCACTGACCACTTGAG | AGCCCCCAGACTTACCTTCT |
| AK138493 | CTCTCTCGAATGCACGTCCG | GTGACCCTTACCCATCCATCC |
| ENSMUST00000098305 | CCCAAACTGGCATTGTTCCC | CTGCCTGCCCCTACAAACAT |
| β-actin | ACCTTCTACAATGAGCTGCG | CTGGATGGCTACGTACATGG |

**Supplementary Table 3. Top 10 differentially expressed lncRNAs in IR-EVs VS S-EVs.**

|  | **LncRNA ID** | **Fold change** | **P Value** |
| --- | --- | --- | --- |
| Down-regulated | TCONS_00006932 | 3.775113 | 0.0146 |
|  | AK041430 | 3.38785 | 0.00135 |
|  | TCONS_00013489 | 3.294821 | 0.0432 |
|  | NR_037589 | 2.963866 | 0.02395 |
|  | NR_037589 | 2.963866 | 0.02395 |
| Up-regulated | TCONS_00010866 | 45.6971 | 0.00025 |
|  | ENSMUST00000098305 | 41.8519 | 0.03565 |
|  | TCONS_00025153 | 5.11413 | 0.0053 |
|  | uc029sug.1 | 5.01215 | 0.0127 |
|  | AK028038 | 4.07209 | 0.03855 |

**Supplementary Table 4. Potential cis-target genes of the differentially expressed** **lncRNAs**

| **LncRNA** | **Regulation** | **classification** | **Target gene** | **Target gene** | |
| --- | --- | --- | --- | --- | --- |
|  |  |  |  | **Start** | **End** |
| 2_00008059 | up | intronic antisense | Jarid2 | 44730837 | 44903258 |
| 2_00008059 | up | intronic antisense | Jarid2 | 44840690 | 44914319 |
| 2_00008059 | up | intronic antisense | Jarid2 | 44731270 | 44921643 |
| 2_00008059 | up | intronic antisense | Jarid2 | 44730773 | 44921643 |
| 2_00008059 | up | intronic antisense | Jarid2 | 44730773 | 44921643 |
| 2_00014852 | up | intergenic |  |  |  |
| 2_00033909 | up | intergenic |  |  |  |
| AK009210 | up | intergenic |  |  |  |
| AK014131 | up | bidirectional | Pde7a | 19223107 | 19311322 |
| AK028038 | up | intergenic |  |  |  |
| AK035396 | up | intergenic |  |  |  |
| AK037257 | up | intergenic |  |  |  |
| AK039043 | up | intergenic |  |  |  |
| AK041306 | up | intergenic |  |  |  |
| AK077177 | up | intergenic |  |  |  |
| AK082217 | up | intergenic |  |  |  |
| AK083183 | up | intergenic |  |  |  |
| AK138493 | up | intergenic |  |  |  |
| AK143604 | up | bidirectional | Cdkl4 | 80523549 | 80563834 |
| AK156559 | up | intergenic |  |  |  |
| ENSMUST00000098305 | up | bidirectional | Rab30 | 92741713 | 92837117 |
| ENSMUST00000123700 | up | intergenic |  |  |  |
| ENSMUST00000144604 | up | intergenic |  |  |  |
| ENSMUST00000151051 | up | intergenic |  |  |  |
| NR_015519 | up | intergenic |  |  |  |
| NR_015614 | up | exon sense-overlapping | E230029C05Rik | 90029158 | 90049069 |
| NR_033139 | up | exon sense-overlapping | AI427809 | 53261892 | 53262547 |
| NR_033498 | up | intergenic |  |  |  |
| TCONS_00004004 | up | intergenic |  |  |  |
| TCONS_00009539 | up | exon sense-overlapping | Tcrd | 54122273 | 54146163 |
| TCONS_00009539 | up | exon sense-overlapping | Tcrd | 54136777 | 54146163 |
| TCONS_00009555 | up | intergenic |  |  |  |
| TCONS_00010866 | up | intergenic |  |  |  |
| TCONS_00013490 | up | intergenic |  |  |  |
| TCONS_00025153 | up | intergenic |  |  |  |
| TCONS_00025431 | up | intergenic |  |  |  |
| TCONS_00029481 | up | intergenic |  |  |  |
| TCONS_00034155 | up | intergenic |  |  |  |
| uc007pou.1 | up | intergenic |  |  |  |
| uc007vxz.1 | up | intergenic |  |  |  |
| uc007zhs.2 | up | exon sense-overlapping | Cd200r1 | 44765735 | 44794977 |
| uc008uzu.1 | up | exon sense-overlapping | Laptm5 | 130915948 | 130935078 |
| uc008uzu.1 | up | exon sense-overlapping | Laptm5 | 130913307 | 130928669 |
| uc008uzu.1 | up | exon sense-overlapping | Laptm5 | 130913357 | 130933666 |
| uc008uzu.1 | up | exon sense-overlapping | Laptm5 | 130913333 | 130936148 |
| uc008uzu.1 | up | exon sense-overlapping | Laptm5 | 130913333 | 130935157 |
| uc009qri.1 | up | exon sense-overlapping | Fam214a | 74953052 | 75032468 |
| uc029sug.1 | up | intergenic |  |  |  |
| AA870446_1 | down | intergenic |  |  |  |
| AK030286 | down | bidirectional | Etf1 | 34931833 | 34931941 |
| AK030286 | down | bidirectional | Etf1 | 34902784 | 34932003 |
| AK035001 | down | intergenic |  |  |  |
| AK041430 | down | intergenic |  |  |  |
| AK047584 | down | intergenic |  |  |  |
| AK049862 | down | intergenic |  |  |  |
| AK054291 | down | intergenic |  |  |  |
| AK081948 | down | intergenic |  |  |  |
| AK089363 | down | intergenic |  |  |  |
| AK133488 | down | intergenic |  |  |  |
| AK136499 | down | intergenic |  |  |  |
| AK142369 | down | bidirectional | Cherp | 72460482 | 72475233 |
| AK142369 | down | bidirectional | Cherp | 72462176 | 72475233 |
| AK142369 | down | bidirectional | Cherp | 72473971 | 72475233 |
| AK142657 | down | intergenic |  |  |  |
| AK145174 | down | intergenic |  |  |  |
| AK149597 | down | intergenic |  |  |  |
| ENSMUST00000146010 | down | intergenic |  |  |  |
| ENSMUST00000174924 | down | bidirectional | Rps29 | 69157721 | 69159186 |
| ENSMUST00000176296 | down | bidirectional | Cct8 | 87483987 | 87495788 |
| ENSMUST00000176296 | down | bidirectional | Cct8 | 87488930 | 87495859 |
| ENSMUST00000176296 | down | bidirectional | Cct8 | 87490487 | 87495704 |
| ENSMUST00000176296 | down | bidirectional | Cct8 | 87483324 | 87495869 |
| ENSMUST00000180630 | down | intergenic |  |  |  |
| ENSMUST00000181422 | down | bidirectional | Slc3a2 | 8706881 | 8723369 |
| ENSMUST00000181621 | down | intergenic |  |  |  |
| NR_027923 | down | bidirectional | Tle1 | 72117804 | 72200857 |
| NR_027923 | down | bidirectional | Tle1 | 72117141 | 72200919 |
| NR_027923 | down | bidirectional | Tle1 | 72117806 | 72200854 |
| NR_027923 | down | bidirectional | Tle1 | 72157579 | 72200862 |
| NR_027923 | down | bidirectional | Tle1 | 72117141 | 72200919 |
| NR_027923 | down | bidirectional | Tle1 | 72117141 | 72200919 |
| NR_027923 | down | bidirectional | Tle1 | 72157580 | 72200919 |
| NR_027923 | down | bidirectional | Tle1 | 72117141 | 72200919 |
| NR_037589 | down | exon sense-overlapping | Rbm7 | 48488696 | 48495330 |
| NR_037589 | down | exon sense-overlapping | Rbm7 | 48488700 | 48495330 |
| NR_037589 | down | exon sense-overlapping | Rbm7 | 48488696 | 48494181 |
| NR_037589 | down | exon sense-overlapping | Rbm7 | 48488696 | 48495330 |
| NR_037589 | down | exon sense-overlapping | Rbm7 | 48488696 | 48495330 |
| NR_045042 | down | intergenic |  |  |  |
| TCONS_00000487 | down | bidirectional | Ivns1abp | 1.51E+08 | 1.51E+08 |
| TCONS_00000487 | down | bidirectional | Ivns1abp | 1.51E+08 | 1.51E+08 |
| TCONS_00000487 | down | bidirectional | Ivns1abp | 1.51E+08 | 1.51E+08 |
| TCONS_00006671 | down | intron sense-overlapping | B430203G13Rik | 17924293 | 17925668 |
| TCONS_00006932 | down | intergenic |  |  |  |
| TCONS_00013489 | down | intergenic |  |  |  |
| TCONS_00015067 | down | intergenic |  |  |  |
| uc007cyp.1 | down | exon sense-overlapping | Ivns1abp | 1.51E+08 | 1.51E+08 |
| uc007cyp.1 | down | exon sense-overlapping | Ivns1abp | 1.51E+08 | 1.51E+08 |
| uc007cyp.1 | down | exon sense-overlapping | Ivns1abp | 1.51E+08 | 1.51E+08 |
| uc007hxv.1 | down | intronic antisense | Camk2b | 5971671 | 6065742 |
| uc007hxv.1 | down | intronic antisense | Camk2b | 5971679 | 6065612 |
| uc007hxv.1 | down | intronic antisense | Camk2b | 5971671 | 6065545 |
| uc007hxv.1 | down | intronic antisense | Camk2b | 5971668 | 6065582 |
| uc007hxv.1 | down | intronic antisense | Camk2b | 5971734 | 6065538 |
| uc007hxv.1 | down | intronic antisense | Camk2b | 5972216 | 6065570 |
| uc007hxv.1 | down | intronic antisense | Camk2b | 5969671 | 6065563 |
| uc007hxv.1 | down | intronic antisense | Camk2b | 5969665 | 6065748 |
| uc007hxv.1 | down | intronic antisense | Camk2b | 5969665 | 6065748 |
| uc007hxv.1 | down | intronic antisense | Camk2b | 5969665 | 6065748 |
| uc008gpa.1 | down | intergenic |  |  |  |
| uc008shx.1 | down | intergenic |  |  |  |
| uc009hcl.2 | down | exon sense-overlapping | Svip | 51997160 | 52006018 |

**Supplementary Table 5. Top 10 differentially expressed mRNAs in IR-EVs VS S-EVs.**

|  | **mRNA ID** | **Fold change** | **P Value** |
| --- | --- | --- | --- |
| Down-regulated | Defa2 | 20.98345 | 0.00005 |
|  | Ddah1 | 7.465506 | 0.01975 |
|  | Hemt1 | 6.416649 | 0.0052 |
|  | Gm6710 | 6.251793 | 0.03765 |
|  | 5730507C01Rik | 5.368876 | 0.00005 |
| Up-regulated | Bcmo1 | 14.97 | 0.00005 |
|  | Mcpt1 | 9.05108 | 0.0019 |
|  | Lars2 | 9.01089 | 0.00005 |
|  | Hp | 8.97318 | 0.00005 |
|  | Xlr4c | 6.40443 | 0.01015 |

**Supplementary Table 6. Results of linear correlation analysis**

|  | ENSMUST00000146010 | ENSMUST00000180630 | TCONS_00010866 |
| --- | --- | --- | --- |
| **AST** |  |  |  |
| R^2^ | 0.5198 | 0.5742 | 0.5418 |
| P value | 0.0186 | 0.0111 | 0.0152 |
| Equation | Y = -537.0*X + 781.0 | Y = -642.6*X + 846.5 | Y = 474.2*X - 248.1 |
| **LDH** |  |  |  |
| R^2^ | 0.6037 | 0.6854 | 0.6693 |
| P value | 0.0082 | 0.0031 | 0.0038 |
| Equation | Y = -2249*X + 2738 | Y = -2728*X + 3039 | Y = 2048*X - 1653 |
| **CK** |  |  |  |
| R^2^ | 0.5429 | 0.8881 | 0.6861 |
| P value | 0.0151 | <0.0001 | 0.0031 |
| Equation | Y = -6218*X + 7382 | Y = -9055*X + 9319 | Y = 6133*X - 5372 |
| **CK-MB** |  |  |  |
| R^2^ | 0.2791 | 0.08584 | 0.2973 |
| P value | 0.1164 | 0.4113 | 0.1031 |
| Equation | Y = -528.4*X + 913.2 | Y = -333.6*X + 759.6 | Y = 471.6*X - 106.1 |
| **EF** |  |  |  |
| R^2^ | 0.4317 | 0.5932 | 0.6018 |
| P value | 0.0390 | 0.0091 | 0.0084 |
| Equation | Y = 38.24*X + 11.34 | Y = 51.04*X + 2.819 | Y = -39.05*X + 91.59 |
| **FS** |  |  |  |
| R^2^ | 0.4197 | 0.5676 | 0.5923 |
| P value | 0.0428 | 0.0119 | 0.0092 |
| Equation | Y = 20.87*X + 3.833 | Y = 27.63*X - 0.6517 | Y = -21.44*X + 47.79 |

AST, aspartate transaminase; LDH, lactate dehydrogenase; CK, creatine kinase; CK-MB, creatine kinase isoenzyme; EF, ejection fraction; FS, fraction shortening.
